# Simultaneous Removal of Heavy Metals and Dyes on Sodium Alginate/Polyvinyl Alcohol/κ-Carrageenan Aerogel Beads

**DOI:** 10.3390/gels11030211

**Published:** 2025-03-16

**Authors:** Taesoon Jang, Soyeong Yoon, Jin-Hyuk Choi, Narae Kim, Jeong-Ann Park

**Affiliations:** Department of Environmental Engineering, Kangwon National University, Chuncheon 24341, Republic of Korea; zxotnsdlz@kangwon.ac.kr (T.J.); sy3436@kangwon.ac.kr (S.Y.); cjh0722@kangwon.ac.kr (J.-H.C.); narae345@kangwon.ac.kr (N.K.)

**Keywords:** sodium alginate, polyvinyl alcohol, κ-carrageenan, semi-interpenetrating polymer network, aerogel bead, heavy metal, dye

## Abstract

Industrial textile wastewater containing both heavy metals and dyes has been massively produced. In this study, semi-interpenetrating polymer network structures of sodium alginate (SA)/polyvinyl alcohol (PVA)/κ-carrageenan (CG) aerogel beads were synthesized for their simultaneous reduction. The SA/PVA/CG aerogel beads were synthesized through a cost-effective and environmentally friendly method using naturally abundant biopolymers without toxic cross-linkers. The SA/PVA/CG aerogel beads were spheres with a size of 3.8 ± 0.1 mm, exhibiting total pore areas of 15.2 m^2^/g and porous structures (pore size distribution: 0.04–242.7 μm; porosity: 93.97%) with abundant hydrogen bonding, high water absorption capacity, and chemical resistance. The adsorption capacity and mechanisms of the SA/PVA/CG aerogel beads were investigated through kinetic and isotherm experiments for heavy metals (Cu(II), Pb(II)), cationic dye (methylene blue, MB), and anionic dye (acid blue 25, AB)) in both single and binary systems. The maximum adsorption capacities of the SA/PVA/CG aerogel beads based on the Langmuir model of Cu(II), Pb(II), and MB were 85.17, 265.98, and 1324.30 mg/g, respectively. Pb(II) showed higher adsorption affinity than Cu(II) based on ionic properties, such as electronegativity and hydration radius. The adsorption of Cu(II), Pb(II), and MB on the SA/PVA/CG aerogel beads was spontaneous, with heavy metals and MB exhibiting endothermic and exothermic natures, respectively.

## 1. Introduction

The textile industry is a significant consumer of water, leading to the generation of large volumes of wastewater containing both dye and heavy metal pollutants [1]. Dyes, which are widely used in paper, leather, fiber, and rubber manufacturing, are non-biodegradable and recalcitrant due to their complex structures [2,3], while heavy metals, such as copper (Cu) and lead (Pb), are employed to produce the color pigments for textile dyes [4]. To effectively remove these heavy metals and dyes from industrial wastewater, various physical and chemical treatments have been developed, including chemical precipitation, ion exchange, adsorption, and electrochemical treatment [5,6]. Of these methods, adsorption is the most common due to its low establishment costs, simple design, and ease of operation [7,8].

However, when heavy metals and dyes are present together in wastewater, their interactions can complicate the adsorption process [9,10,11,12]. This is because the formation of complexes introduces coordination and steric effects, while competition for adsorption sites or bridging effects can also occur. Therefore, it is necessary to compare the adsorption mechanisms in binary-pollutant systems with those in single-pollutant systems and develop more effective strategies for the effective simultaneous removal of heavy metals and dyes. In line with this, several studies have developed adsorbents for use in these binary systems based on activated carbon [13], carbon nanotubes [14], iron oxides’ nanoparticles [15], layered double hydroxides [16], and biomass-incorporating composites [17]. Although these adsorbents show demonstrated promise in removing heavy metals and dyes in binary systems, the spent adsorbent, which includes inorganic nanomaterials and/or carbon materials, becomes a form of secondary waste that requires an additional disposal process to ensure that it does not pose potential environmental risks [18].

A number of recent studies have reported the synthesis of adsorbents based on environmentally friendly biopolymers using green synthesis methods as a means to reduce the environmental risks and protect public health. For example, sodium alginate (SA) is an inexpensive and natural polysaccharide polymer extracted from algae [19] that possesses abundant carboxyl (–COOH) and hydroxyl (–OH) groups, making it suitable for cationic heavy metal adsorption [20]. ĸ-carrageenan (CG) is another useful polysaccharide polymer with a high number of hydroxyl and sulfate groups (–OSO_3_⁻) [21]. Although SA and CG can be cross-linked using Ca(II) [19], they have low physical and chemical resistance [22,23]. Alginate was susceptible to hydrolysis or degradation in strong acid and alkali solution, and sulfate groups on CG dissociate more readily than carboxylate groups on alginate [19,24]. Yu et al. [22] confirmed that CG/SA hydrogel beads shows a high swelling rate. On the other hand, polyvinyl alcohol (PVA) is a non-toxic and biocompatible synthetic polymer with high chemical resistance, remaining stable in pH 1–13 [25] and also offering numerous hydroxyl groups [26]. Due to these complementary advantages, the removal of heavy metals or dyes using composite adsorbents containing SA, CG, and PVA has been widely investigated. Croitoru et al. [27] synthesized an SA/PVA hydrogel with high stability in solutions with various ionic strengths and pH levels that exhibited a maximum adsorption capacity for crystal violet of 121.4 mg/g. Radoor et al. [28] also reported a PVA/CG membrane with a methylene blue (MB) adsorption capacity of 147.8 mg/g, the highest of various carrageenan-based adsorbents. However, no previous research has been conducted to remove both heavy metals and dyes simultaneously with a composite based on an SA, PVA, and CG polymer networks.

In a semi-interpenetrating polymer network (semi-IPN), a cross-linked polymer and a linear polymer are interlaced and entangled [29]. Because one of the polymers is not cross-linked, functional groups, such as hydroxyl groups, can participate in the adsorption of pollutants, while the interlaced and entangled network improves the mechanical strength. Although chemically cross-linked pure hydrogels are inflexible and can easily break when in a swollen state, PVA can effectively transfer stress through non-covalent bonding with other polymers, improving the performance of polymer networks [30,31]. Kim and Min synthesized a semi-IPN with calcium alginate and a PVA hydrogel that demonstrated two to five times greater mechanical and physiochemical stability compared to a pure alginate hydrogel [32]. Although the semi-IPN structure preserves the distinctive characteristics of polymers while alleviating their weakness, to the best of our knowledge, the development and testing of a semi-IPN structure containing environmentally friendly and cost-effective polymers (SA, CG, and PVA) for adsorption of pollutants has not yet been reported. Also, aerogel has several advantages over hydrogel, such as its high pore area, rapid adsorption rate, and light weight. In addition, although many adsorption studies have investigated heavy metals and dyes in mono-polluted systems, no previous research has investigated the potential simultaneous removal of two pollutants in wastewater. Therefore, the objectives of the present study were to (1) characterize SA-, CG-, and PVA-based semi-IPN aerogel bead types of adsorbents synthesized using an environmentally friendly method, (2) compare the adsorption capacities of these adsorbents in single-pollutant systems (Cu(II), Pb(II), and MB or acid blue 25(AB)) and verify the associated adsorption mechanisms, (3) investigate the removal capacity of the adsorbents in binary-pollutant systems, and (4) evaluate their acute toxicity with *Daphnia magna (D. magna)* and biodegradability in soil.

## 2. Results and Discussion

### 2.1. Comparison of the Characteristics and Adsorption Capacity of the SA-Based Aerogel Beads

**Figure 1:**
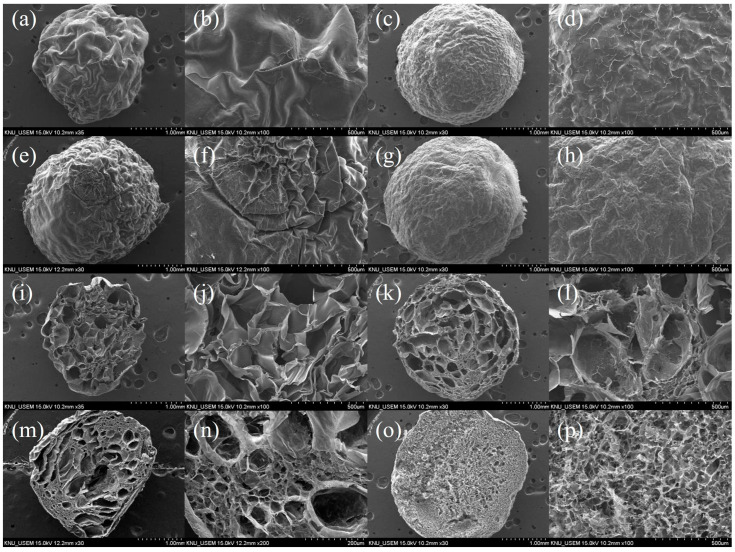
FE-SEM images of surface (**a**,**b**) SA, (**c**,**d**) SA/PVA, (**e**,**f**) SA/CG, and (**g**,**h**) SA/PVA/CG beads and cross-section images of (**i**,**j**) SA, (**k**,**l**) SA/PVA, (**m**,**n**) SA/CG, and (**o**,**p**) SA/PVA/CG beads.

FE-SEM images of the surface and cross-section of the SA, SA/PVA, SA/PVA, and SA/PVA/CG aerogel beads are presented in Figure 1. All beads were spherical in shape owing to the drop-wise synthesis procedure. The SA aerogel beads had a crumbled surface (Figure 1a,b), while the other aerogel beads exhibited a spherical shape with a relatively smooth surface (Figure 1c–h). As shown in Figure 1, the introduction of both PVA and CG to SA resulted in a densely porous structure with a uniform distribution of smaller pores. Due to the rapid cross-linking of the outer surface of the SA/PVA/CG aerogel beads, small pores formed close to the bead’s surface, with pore sizes gradually increasing towards the interior. The size of the SA/PVA/CG aerogel beads is 3.8 ± 0.1 mm, and the total pore area of 15.2 m^2^/g. The pore sizes of the SA/PVA/CG aerogel beads ranged from 0.04 to 242.7 μm, with an average diameter of 8.9 μm and a porosity of 73.97%.

**Figure 2:**
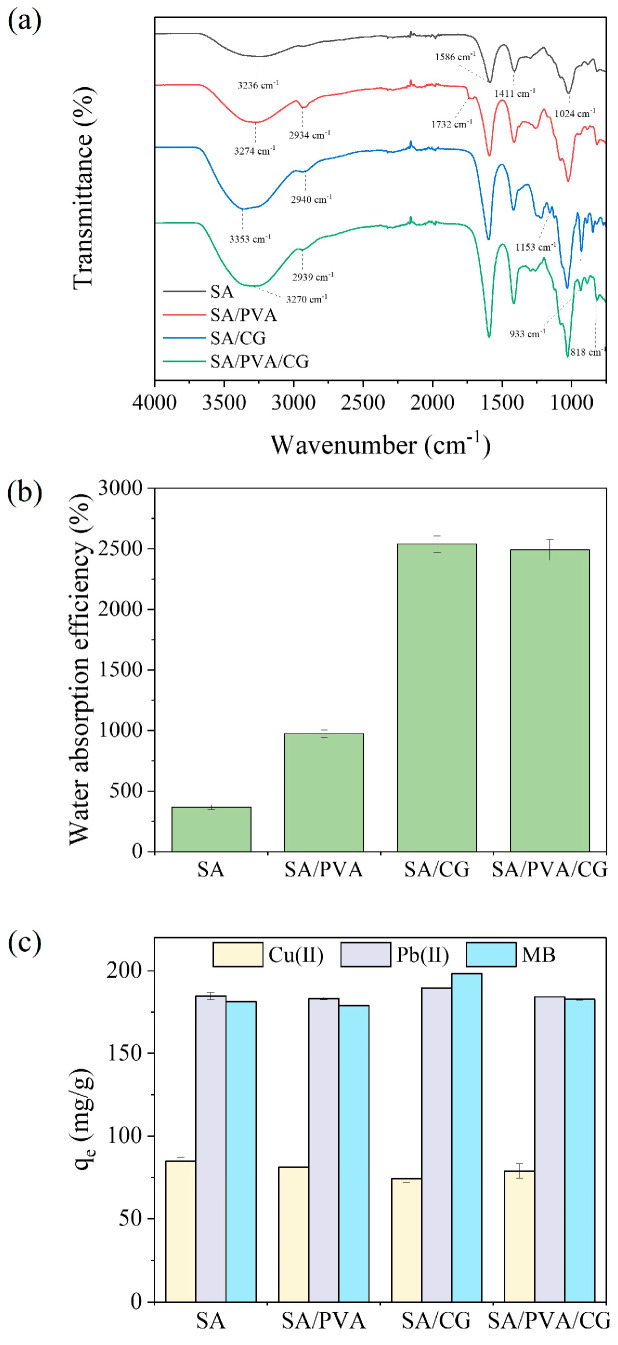
Characteristics of the SA/PVA, SA/CG, and SA/PVA/CG aerogel beads: (**a**) FT-IR spectra, (**b**) water absorption efficiency, (**c**) and adsorption capacity for Cu(II), Pb(II), and MB.

Figure 2a displays the FTIR spectra for the SA, SA/PVA, SA/CG, and SA/PVA/CG aerogel beads. All of the beads exhibited peaks from 3000 to 3600 cm^−1^, corresponding to the stretch vibrations of the –OH groups. The peaks observed at 1586 cm^−1^ and 1411 cm^−1^ for the SA aerogel beads were ascribed to asymmetric and symmetric O-C=O stretching vibrations [31], while the peak at 1024 cm^−1^ indicated the presence of –C-O-C groups [33]. In the spectra for the SA/PVA aerogel beads, the addition of PVA produced a new peak at 1732 cm^−1^, corresponding to C=O stretching vibrations from the residual ester groups of PVA. It was confirmed that PVA was not cross-linked in SA/PVA aerogel beads, providing an additional –OH group. In the SA/CG and SA/PVA/CG aerogel beads, peaks of 1153 cm^−1^, 933 cm^−1^, and 818 cm^−1^ were assigned to ester sulfate -S=O stretching, 3, 6-anhydro-D-galactose, and ester sulfate stretching, respectively [27,34,35]. The SA/PVA/CG aerogel beads exhibited the strongest peak for the –OH groups, indicating that it could potentially have higher pollutant adsorption. These results indicated that SA/PVA/CG aerogel beads had high density due to hydrogen bonding (Figure 1).

The water absorption capacity of the SA-based aerogel beads is summarized in Figure 2b. All of the beads were buoyant in water due to the presence of large pores and their low density. Due to the high hydrophilicity of CG, the SA/CG (2539.86 ± 68.57%) and SA/PVA/CG (2492.76 ± 87.69%) aerogel beads demonstrated high water absorption capacity. The SA/PVA/CG aerogel beads showed slightly lower water absorption capacity than that of SA/CG beads due to containing less CG, which has higher hydrophilicity than PVA. However, the value is still very high, confirming high hydrophilicity. The high water absorption affinity was related to the flexibility of the porous network, which facilitated expansion and, potentially, the rapid diffusion of pollutants, thus increasing the number of contact sites [36,37]. The Cu(II), Pb(II), and MB adsorption capacities for the SA-based beads are presented in Figure 2c. SA aerogel beads exhibited the highest Cu(II) adsorption capacity at 84.8 ± 2.45 mg/g, while the SA/CG aerogel beads had the highest Pb(II) and MB adsorption capacity (189.49 ± 0.13 and 198.30 ± 0.12 mg/g, respectively). However, all of the SA-based aerogel beads had a similar adsorption capacity for Cu(II) (74.04–84.80 mg/g), Pb(II) (183.15–189.49 mg/g), and MB (178.81–198.3 mg/g), which indicated that incorporation of the polymers maintained the initial adsorption capacity of SA while also introducing the useful characteristics of the polymers.

The chemical stability of the SA, SA/PVA, SA/CG, and SA/PVA/CG aerogel beads was also evaluated in soaking experiments using both acidic (pH 2) and basic (pH 9) solutions. All of the aerogel beads were buoyant in the initial stages of the experiment; however, the SA aerogel beads in the pH 2 solution and the SA, SA/PVA, and SA/CG aerogel beads in the pH 9 solution gradually settled on the bottom of the container after 10 days of exposure. The SA aerogel beads crumbled and shrank under both acidic (Figure S1a–c) and basic (Figure S1d–f) conditions. Chen et al. [38] also reported that alginate is prone to shrinking at low pH. As shown in Figure S1g–i, the SA, SA/PVA, and SA/CG aerogel beads in the basic solution also exhibited deformation and the expansion of their pore networks, possibly due to swelling in the weak basic solution, followed by erosion and disintegration [39]. Only the SA/PVA/CG aerogel beads maintained their buoyancy in both the acidic and basic solutions, likely due to hydrogen bonding interactions between the three types of polymers. Because the SA/PVA/CG aerogel beads exhibited the strongest chemical stability under extreme pH conditions, they were used for further analysis.

### 2.2. Adsorption Mechanisms for the SA/PVA/CG Aerogel Beads in Single-Pollutant Removal

#### 2.2.1. Adsorption Kinetics

**Figure 3:**
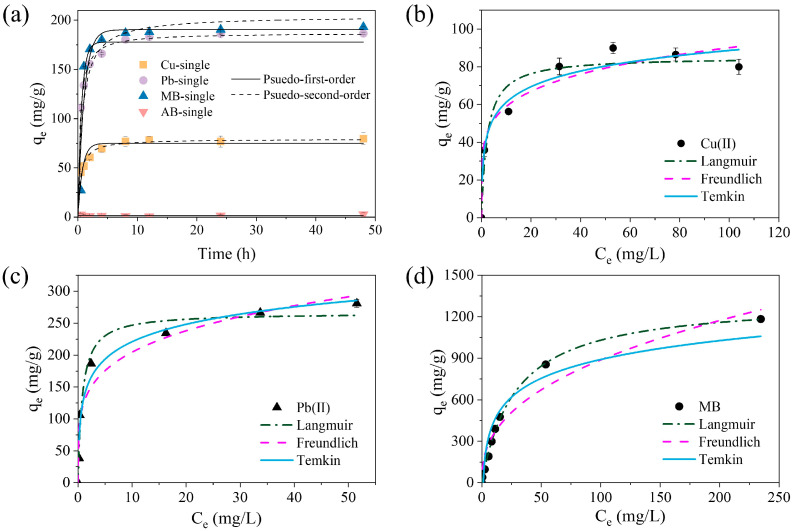
Adsorption of single pollutants (Cu(II), Pb(II), MB, or AB) onto the SA/PVA/CG aerogel beads: (**a**) kinetic and isotherm data for (**b**) Cu(II) (C_0_ = 10–125 mg/L), (**c**) Pb(II) (C_0_ = 10–125 mg/L), and (**d**) MB (C_0_ = 10–500 mg/L).

The effect of the reaction time on the adsorption of single pollutants (Cu(II), Pb(II), MB, and AB) using the SA/PVA/CG aerogel beads was evaluated (Figure 3a). The adsorption capacity of Cu (II), Pb(II), and MB rapidly increased over the initial 4 h and reached an adsorption equilibrium before 24 h had passed. The adsorption kinetic parameters for each pollutant using the SA/PVA/CG aerogel beads are listed in Table S1. The adsorption kinetic data for Cu(II) and Pb(II) fit the pseudo-second-order model well, indicating that chemical adsorption was the main rate-controlling step [40]. The adsorption capacity (q_e_) and rate constant (k_2_) for Pb(II) were higher than those for Cu(II), suggesting that the adsorption of Pb(II) occurred more rapidly and may be preferred on the SA/PVA/CG aerogel beads. Generally, aerogels exhibit rapid adsorption rates due to their open porous structure filled with air and high surface area, enhancing the fast diffusion of heavy metals into the interior of the beads. The beads exhibited a greater adsorption affinity for Pb(II) than for Cu(II) because Pb(II) has greater electronegativity (2.33) and a smaller hydration radius (4.01 Å), leading to stronger electrostatic interactions with the SA/PVA/CG aerogel beads [41,42].

The adsorption of MB more closely followed the pseudo-first-order model (R^2^ = 0.912) than the pseudo-second-order model (R^2^ = 0.873). This indicates that MB adsorption was likely due to physical adsorption [43]. On the other hand, the adsorption of AB onto the SA/PVA/CG aerogel beads was insignificant after 48 h. This could be explained by the negatively charged surface of the SA/PVA/CG aerogel beads (zeta potential = −26.9 ± 0.4 mV at pH 5.7; Figure S2), which repelled the anionic dye AB. Because of this lack of adsorption, AB was excluded from further single-pollutant adsorption experiments.

#### 2.2.2. Adsorption Isotherms

As shown in Figure 3, the adsorption isotherm data for the single pollutants (Cu(II), Pb(II), and MB) using the SA/PVA/CG aerogel beads were fitted using the Langmuir, Freundlich, and Temkin models. The parameters for the isotherm experiments are summarized in Table S2. The results indicated that the adsorption of Cu(II) and Pb(II) most closely followed the Temkin model. This means that multilayer adsorption was dominant, with a uniform binding energy distribution on the SA/PVA/CG aerogel beads up to the maximum binding energy [44]. The adsorption of MB was more accurately described by the Langmuir model (R^2^ = 0.982) than the Freundlich (R^2^ = 0.937) and Temkin models (R^2^ = 0.876). This suggested that the main adsorption mechanism for MB was monolayer adsorption [45]. In addition, the b_T_ of Cu(II), Pb(II), and MB was positive, suggesting that the adsorption process was an endothermic reaction [46]. The maximum adsorption capacity (q_m_) of Cu(II), Pb(II), and MB on the SA/PAV/CG aerogel beads was determined to be 85.17, 265.98, and 1324.30 mg/g, respectively, using the Langmuir model. The 1/n values for the Freundlich model (Cu(II) = 0.18; Pb(II) = 0.22; and MB = 0.40) were lower than 1, indicating that the adsorption of Cu(II), Pb(II), and MB onto the SA/PVA/CG aerogel beads was favorable [47,48,49].

The maximum adsorption capacities of Cu(II), Pb(II), and MB using SA-, PVA-, and CG-based adsorbents reported in previous studies are presented in Table S3. The adsorption capacity for Cu(II) in these past studies had a range of 29.1–114.94 mg/g, with the highest capacity obtained by incorporating graphene oxide (GO) (98.0–105.93 mg/g) [50,51] or Fe_3_O_4_ (114.94 mg/g) [52]. However, the SA/PVA/CG aerogel beads synthesized in the present study had the highest adsorption capacity (85.17 mg/g) among those adsorbents composed only of polymers (29.19–70.45 mg/g) [53]. The adsorption capacity of the SA/PVA/CG aerogel beads for Pb(II) (265.98 mg/g) was comparable to previously reported adsorbents containing GO (191.94–327.9 mg/g) [51,54], while the SA/PVA/CG aerogel beads had a higher MB adsorption capacity (1324.30 mg/g) than the other reported adsorbents (94.3–628.93 mg/g) [55,56]. The high pore area, porosity, and hydrophilic nature of the SA/PVA/CG aerogel beads provide sufficient adsorption sites for both MB and heavy metals. These results confirmed that the SA/PVA/CG aerogel beads, produced using environmentally friendly materials and chemicals and a simple synthesis method, are a promising adsorbent for the removal of heavy metals and dyes.

#### 2.2.3. Effects of pH and Temperature

**Figure 4:**
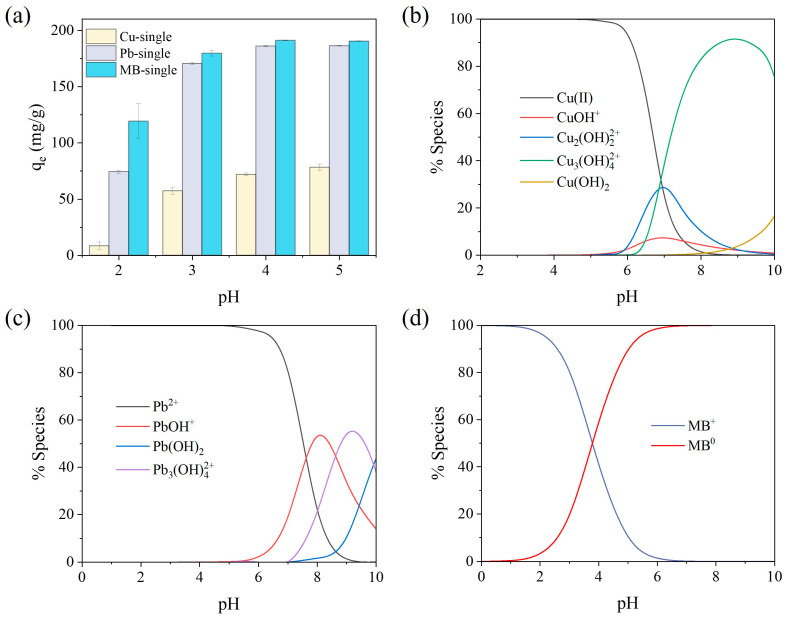
Effect of pH on (**a**) Cu(II), Pb(II), and MB adsorption using the SA/PVA/CG aerogel beads and distribution of (**b**) Cu(II), (**c**) Pb(II), and (**d**) MB.

The pH of a solution can affect the ionization stage of the functional groups in both the SA/PVA/CG aerogel beads and the pollutants, thus influencing the adsorption behavior of the system. The SA/PVA/CG aerogel beads exhibited an increase in adsorption capacity for Cu(II), Pb(II), and MB with an increase in the pH (Figure 4a). The adsorption capacity was the lowest at pH 2 (Cu(II): 8.52 ± 3.18 mg/g; Pb(II): 74.36 ± 1.41 mg/g; MB: 119.21 ± 15.6 mg/g) and the highest at pH 5 (Cu(II): 78.32 ± 2.79 mg/g; Pb(II): 186.35 ± 0.38 mg/g; MB: 190.42 ± 20.18 mg/g). The pH levels up until pH 5 because Cu(II) ions form a Cu(OH)_2_ precipitate at pH ≥ 6 (Figure 4b) [57]. The pH species distribution for Cu(II), Pb(II), and MB is presented in Figure 4b–d, showing that Cu and Pb were present in an ionic state as Cu(II) and Pb(II) at pH levels below 5, while MB was strongly positive with fully protonated amine groups at a pH lower than 3.8 (pKa) and present as MB^+^ at a pH of over 3.8 [58,59].

As shown in Figure S2, the zeta potential of the SA/PVA/CG aerogel beads was negative from pH 2 to 5.8 and became more negative as the pH increased, resulting in a stronger adsorption capacity for the cationic pollutants (Cu(II), Pb(II), and MB). In addition, at higher pH levels, the proportion of neutral MB (MB0) increases, leading to stronger non-electrostatic interactions, such as hydrophobic interactions [60]. On the other hand, at low pH levels, the adsorption capacity for Cu(II), Pb(II), and MB decreased because the adsorption sites on the SA/PVA/CG aerogel beads were partially protonated, weakening the electrostatic interaction between the negatively charged aerogel beads and the cationic pollutants.

**Figure 5:**
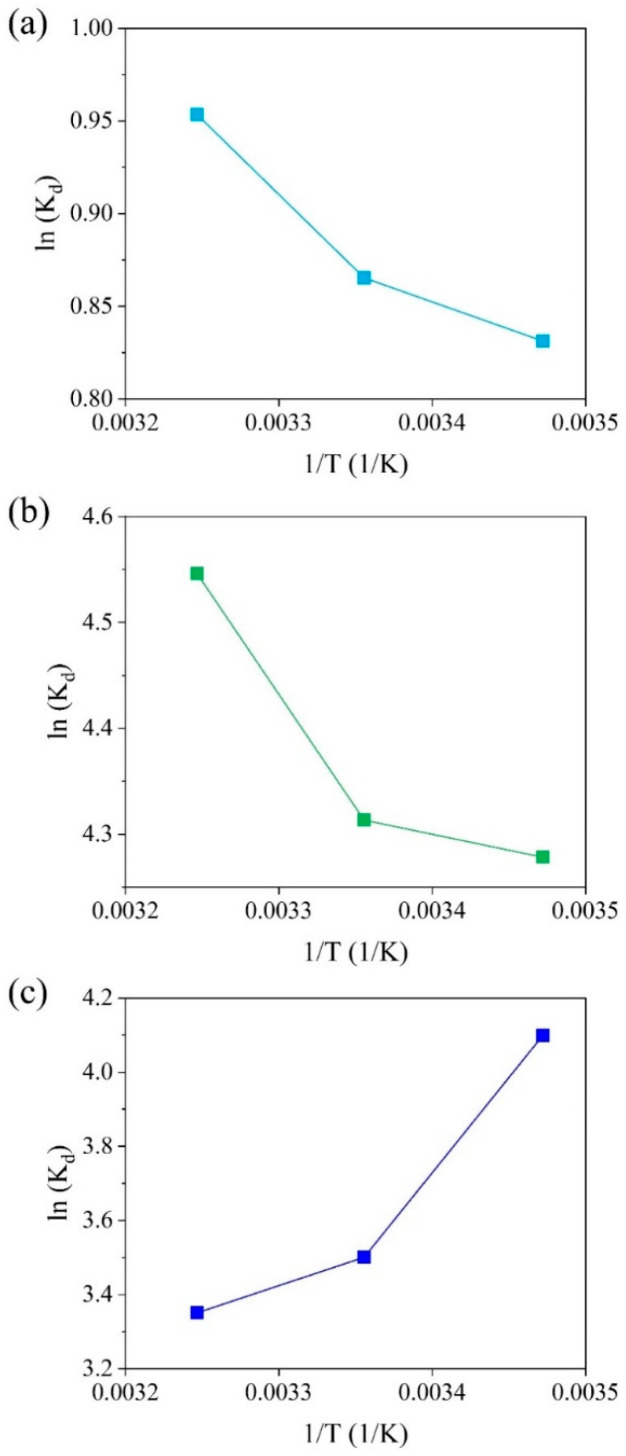
Effect of temperature on (**a**) Cu(II), (**b**) Pb(II), and (**c**) MB adsorption using the SA/PVA/CG aerogel beads.

A thermodynamic analysis was conducted to investigate the spontaneity and thermodynamic properties of the adsorption process. The effect of temperature was tested at 288, 298, and 308 K (Figure 5), and the thermodynamic parameters for the adsorption of the individual pollutants (Cu(II), Pb(II), or MB) on the SA/PVA/CG aerogel beads are presented in Table S4. For Cu(II) and Pb(II), the negative ΔG^0^ (Cu(II) = −1.96 to −2.41 KJ/mol; Pb(II) = −10.16 to −11.55 KJ/mol) and the positive ΔH^0^ (Cu(II) = 4.48 KJ/mol; Pb(II) = 9.79 KJ/mol) indicated that their adsorption was spontaneous and endothermic. In addition, a positive ΔH^0^ is suggestive of a chemical adsorption process, while higher values indicate the possibility of stronger bonding between the adsorbent and the adsorbate [61]. Therefore, a higher adsorption affinity for Pb(II) than for Cu(II) was observed, which was in correspondence with the adsorption isotherm and kinetic data. The positive values of ΔS^0^ for the adsorption of Cu(II) (22.36 J/mol·K) and Pb(II) (69.29 J/mol·K) indicated that the randomness increased at the solid–liquid interface during the adsorption process. Positive values of ΔH^0^ and ΔS^0^ when cationic heavy metals, including Cu(II) and Pb(II), are adsorbed onto SA/PVA-based composites have also been reported by Isawi [61] and Zhang et al. [53].

For MB adsorption, all of the thermodynamic parameters were negative, including ΔG^0^ (−8.38 to −9.64 KJ/mol), ΔH^0^ (−27.78 KJ/mol), and ΔS^0^ (−62.95 J/mol·K). This indicated that the adsorption of MB was spontaneous and exothermic, and randomness at the interface between the MB and the SA/PVA/CG aerogel beads decreased during the adsorption process. Similarly, Mohammed et al. [62] and Almuslem et al. [63] found that MB adsorption onto cellulose-based films and alginate hydrogels, respectively, tended to be exothermic (ΔH^0^ < 0) and demonstrated lower randomness (ΔS^0^ < 0). The decrease in the adsorption capacity for MB as the temperature increase was due to the weaker physical interaction through hydrogen bonding and van der Waals interactions [64].

### 2.3. Adsorption Mechanisms for the SA/PVA/CG Aerogel Beads in Binary-Pollutant Removal

**Figure 6:**
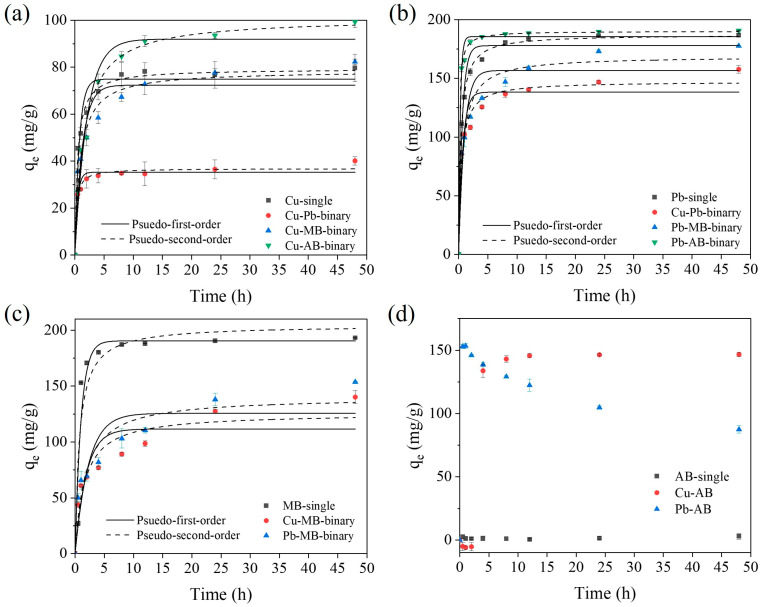
Adsorption kinetic data for single and binary systems using the SA/PVA/CG beads: (**a**) Cu(Ⅱ), (**b**) Pb(Ⅱ), (**c**) MB, and (**d**) AB.

Kinetic adsorption using the SA/PVA/CG aerogel beads in the binary systems (Cu–Pb, Cu–MB, Cu–AB, Pb–MB, and Pb–AB) was tested to compare with the adsorption of the pollutants individually (Figure 6). The calculated kinetic parameters are summarized in Table S5. In the binary systems, the adsorption capacity for Cu(II), Pb(II), and MB increased slightly but continuously, meaning that an equilibrium had not been reached by 48 h (Figure 6a–c). For the adsorption of Cu(II) and Pb(II) in both the single and binary systems, the pseudo-second-order model provided the best fit for the data (R^2^ = 0.980–0.989) than the pseudo-first-order model (R^2^ = 0.948–0.949). This indicated that chemical adsorption was still the dominant adsorption mechanism in the binary systems. However, for MB in the binary systems (Cu–MB and Pb–MB), the pseudo-second-order model led to a better fit (R^2^ = 0.893–0.901), while the pseudo-first-order model was favored for the single MB system. These findings suggested that the adsorption mechanisms have the potential to remain unchanged or vary between single- and binary-pollutant systems.

In the binary Cu–Pb system, the adsorption capacity (q_e_) for both Pb(II) and Cu(II) decreased, suggesting competitive interactions between the heavy metal ions. The q_e_ for Cu(II) (36.87 mg/g) and Pb(II) (147.33 mg/g) decreased by 54.3% and 21.5% compared to their respective single-pollutant systems. However, Pb(II) maintained a higher adsorption affinity than Cu(II). This can be attributed to the higher electronegativity (2.33) and the smaller hydration radius (4.01 Å) of Pb compared to those of Cu (1.90 and 4.19 Å, respectively), resulting in stronger attraction to the negatively charged surface of the SA/PVA/CG aerogel beads and better diffusion into the surface and pores [41]. The k_2_ value (0.11 g/mg/h) for Cu(II) was about four times higher than that in the Cu(II) single-pollutant system (0.03 g/mg/h), while the k_2_ for Pb(II) did not change significantly. This indicates that most of the Cu(II) adsorption occurred quickly but was limited by the competitive effect of Pb(II).

In the binary Cu–MB and Pb–MB systems, the presence of MB did not significantly reduce the adsorption capacity for Pb(II) and Cu(II). However, the MB adsorption capacity in these binary systems was about 61–68% lower than in the single-pollutant MB system (204.53 mg/g). These results suggest that the heavy metals and MB partially competed for adsorption sites on the SA/PVA/CG aerogel beads, with heavy metals exhibiting a stronger adsorption preference for these sites. As described in Section 2.2, the adsorption of Cu(II) and Pb(II) occurred primarily through chemical adsorption mechanisms, while physical adsorption was the dominant mechanism for MB removal. Because of these different adsorption mechanisms between the heavy metals and MB, the total amount of adsorbed pollutants increased despite their competitive effects. Therefore, the SA/PVA/CG aerogel beads could remove the organic dye without inhibiting heavy metal adsorption.

During the adsorption process using the SA/PVA/CG aerogel beads, precipitates were observed in the two binary systems containing AB (Cu–AB and Pb–AB). This precipitate formed instantly after the mixing of the Pb(II) and AB solutions. Generally, Pb(II) naturally forms a precipitate with insoluble sulfates, carbonates, and chloride–hydroxides [65]. Thus, Pb(II) rapidly formed a precipitate with the sulfonate (–SO_3_^−^) of AB. As the reaction time increased, the Pb(II) removal capacity was gradually increased before reaching an equilibrium (Figure 6b), while there was an unusual decrease in the AB removal capacity in the Pb–AB system (Figure 6d). This may have been due to the partial dissociation of the Pb–AB complex when interacting with the released Ca(II) with the addition of the SA/PVA/CG aerogel beads, with the dissociated Pb(II) then adsorbed onto the beads. This was supported by the finding that the Pb’s peak intensity at 1.8 keV was lower and the Ca’s peak intensity at 0.22 keV was stronger after the addition of the SA/PVA/CG aerogel beads (Figure S3a,b).

In the Cu–AB system, no precipitation occurred during the first 2 h of the reaction. However, after this initial period, the AB was removed rapidly due to the formation of a precipitate between the AB and the Ca(II) released from the SA/PVA/CG aerogel beads due to ion exchange via Cu(II). The Ca(II) concentration was found to be 15.1 mg/L after 24 h in the single Cu(II) adsorption reaction. Based on preliminary experiments, Ca(II) and AB (50 mg/L) formed a precipitate when the Ca(II) concentration exceeded 10 mg/L. This finding, along with the observation of a high-intensity peak for Ca in the precipitate (Figure S4c), suggest that the adsorption mechanism for Cu(II) involved ion exchange with Ca(II) in the SA/PVA/CG aerogel beads, and the released Ca(II) subsequently formed a complex with AB. Over time, the release of more Ca(II) led to a greater AB removal capacity. The removal capacity for Cu(II) was also greater than in the Cu system due to both ion exchange and partial complex formation, as confirmed by SEM-EDS data (Figure S3).

### 2.4. Verification of the Adsorption Mechanisms Using XPS Analysis

**Figure 7:**
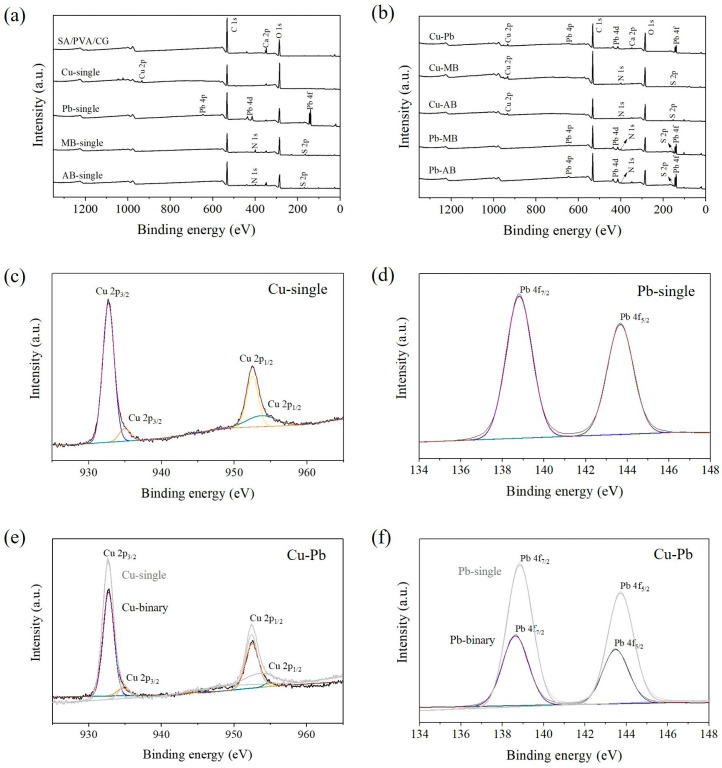
XPS spectra for the SA/PVA/CG aerogel beads (**a**) before and after the adsorption of single pollutants (Cu(II), Pb(II), MB, or AB), (**b**) after the adsorption of two pollutants, (**c**) the Cu 2p region after Cu(II) adsorption, (**d**) the Pb 4f region after Pb(II) adsorption, (**e**) high-resolution version of the Cu 2p region, and (**f**) high-resolution version of the Pb 4f region in the Cu–Pb system.

The XPS spectra for the SA/PVA/CG aerogel beads before and after single-pollutant adsorption (Cu(II), Pb(II), MB, and AB) and binary-pollutant adsorption are presented in Figure 7a. After the adsorption experiment, the beads maintained their morphology well, as shown in Figure S4. The fact that they maintain their shape well suggests a high potential for reusability in future applications. The full spectrum for the unused SA/PVA/CG aerogel beads shows peaks at 164.48, 285.98, 347.24, and 532.17 eV corresponding to S 2p, C 1s, Ca 2p, and O 1s, respectively. The presence of Ca 2p indicated that the SA/PVA/CG aerogel beads were successfully cross-linked by Ca(II). The full-scan XPS spectra for the binary systems are presented in Figure 7b. In the Cu and Pb binary systems, Cu 2p, Pb 4p, Pb 4d, and Pb 4f peaks were also detected, as observed in the single-pollutant system, while in the MB binary systems, N 1s and S 2p peaks appeared for MB. The distinctive XPS spectrum peaks for AB were not vividly observed in the binary systems because the removal of AB was mainly attributed to precipitation with the metal ions. Therefore, these results suggest that Cu(II), Pb(II), and MB were successfully absorbed onto the SA/PVA/CG aerogel beads in the binary systems in a manner similar to the single-pollutant system.

Peaks for Cu 2p_3/2_ and Cu 2p_1/2_ at 932.75 and 952.59 eV, respectively, were present in the Cu single-pollutant system (Figure 7c). The presence of Cu 2p_3/2_ at 932.7 eV and 934.9 eV indicates that the copper oxidation state is Cu(II) [66], meaning that Cu(II) was successfully adsorbed onto the SA/PVA/CG aerogel beads. After Pb(II) adsorption, peaks for Pb 4p (645.5 eV), Pb 4d (414.0 and 436.4 eV), and Pb 4f (138.8 and 143.7 eV) appeared, indicating that Pb was present on the surface of the SA/PVA/CG aerogel beads. As shown in Figure 7d, the presence of peaks for Pb 4f_7/2_ (138.8 eV) and Pb 4f_5/2_ (143.7 eV) was characteristic of Pb(II) species [67], confirming that Pb(II) was coordinated by the SA/PVA/CG beads [68]. In particular, the peak for Pb 4f_7/2_ represented the bonding between Pb(II) and carboxylate groups [69]. The lower energy levels observed for Cu 2p_3/2_ and Cu 2p_1/2_ also confirmed the coordination reaction with the SA/PVA/CG aerogel beads. In the Cu–Pb system, the intensities of the Cu 2p and Pb 4f peaks were significantly lower than those in the respective single-pollutant systems (Figure 7e,f). This indicates that the XPS signals for Cu(II) and Pb(II) on the surface were lower in the Cu–Pb system due to a lower adsorption capacity arising from competitive effects.

**Figure 8:**
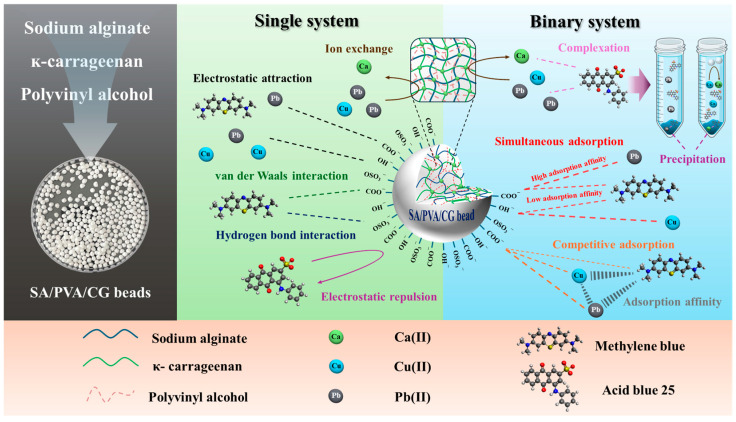
Predicted adsorption mechanism of single and binary system using the SA/PVA/CG aerogel beads.

In the MB single-pollutant system, N 1s peaks appeared at 399.04 and 400.14 eV (Figure S5a), corresponding to the –N(CH_3_)_2_ and –C=N- of MB, respectively [70], thus confirming the successful adsorption of MB onto the SA/PVA/CG aerogel beads. The S 2p peak observed at around 168 eV was attributed to the sulfate group of CG in the SA/PVA/CG aerogel beads (Figure S5b) [71,72]. After the adsorption of MB (Figure S5c), new peaks for S 2p_3/2_ and S 2p_1/2_ appeared at 163.7 and 165.2 eV, respectively, indicating C-S-C in the aromatic ring of MB [73]. Similarly, Zhang et al. [53] also found that S 2p_3/2_ and S 2p_1/2_ peaks appeared after the adsorption of MB. Figure S6 shows the O 1s peaks of the SA/PVA/CG aerogel beads before and after adsorption in the Cu(II), Pb(II), and MB single-pollutant systems. No obvious change in the O 1s peak was observed for MB, indicating that non-electrostatic interactions, such as van der Waals and hydrophobic interactions, were primarily involved in the removal process. These results corresponded well with Section 2.2.1 and Section 2.2.3, which indicated that the adsorption mechanism of MB was dominated by physical adsorption. The predicted adsorption mechanisms of heavy metals and dye in both single and binary systems are presented in Figure 8.

### 2.5. Acute Toxicity and Biodegradability Assessment of the SA/PVA/CG Aerogel Beads

To evaluate the acute toxicity of the SA/PVA/CG aerogel beads, *D. magna* was exposed to various doses of the SA/PVA/CG beads (0.03–0.5 g/L). No immobile *D. magna* individuals were observed after 24 and 48 h of exposure for any of the concentrations. Optical microscopic images of *D. magna* in the control and exposure groups (dose = 0.5 g/L, 48 h) are presented in Figure S7, showing no significant difference between the two. These results indicated that the SA/PVA/CG aerogel beads have no acute toxic effect on *D. magna*. SA and CG are natural polysaccharide-based biopolymers, and PVA is a synthetic polymer that is non-toxic, biocompatible, and biodegradable [20,26,74]. Additionally, only CaCl_2_ was used as an environmentally friendly cross-linker to synthesize the semi-IPN structure of the SA/PVA/CG beads by cross-linking SA and CG, thus avoiding the need for toxic cross-linking agents, such as glutaraldehyde. Therefore, because the constituent polymers are inherently non-toxic and *D. magna* toxicity testing of the composite revealed no toxic effect, the SA/PVA/CG beads are very likely to be safe for use in aquatic ecosystems.

To assess the biodegradability of SA/PVA/CG beads, the weight and FT-IR spectra of the buried beads were measured for 14 days. The weight reduction of SA/PVA/CG beads after 7 and 14 days was 31.65 ± 1.29% and 61.33 ± 1.13%, respectively. FT-IR spectra of the SA/PVA/CG beads before and after biodegradation are presented in Figure S8. The peaks of O-C=O of SA (1586 cm^−1^) and 3,6-anhydro-D-galactose of CG (933 cm^−1^) were distinctively reduced after 14 days. These results indicate that the SA/PVA/CG beads could be biodegraded in natural soil, providing eco-friendly disposal after application for water treatment.

## 3. Conclusions

The SA/PVA/CG aerogel beads were successfully synthesized with a semi-IPN structure using a simple drop-wise procedure. The synthesis utilized naturally abundant biopolymers and required no toxic chemicals. The spherical SA/PVA/CG beads exhibited a densely porous structure, a large pore area, high hydrophilicity, and chemical resistance. These characteristics provided sufficient adsorption sites for heavy metals and dyes, as investigated in both single- and binary-pollutant systems. The adsorption of Cu(II) and Pb(II) was primarily attributed to chemical adsorption via electrostatic interactions and ion exchange, while MB adsorption was dominated by physical adsorption mechanisms, such as van der Waals forces and hydrogen bonding. Due to these distinct mechanisms, the SA/PVA/CG aerogel beads demonstrated the ability to simultaneously adsorb MB without reducing adsorption affinity for Pb(II) and Cu(II). Additionally, the anionic pollutant AB, which was not adsorbed onto the negatively charged SA/PVA/CG aerogel beads in the single-pollutant system, was removed through precipitation in the binary-pollutant system. The synthesized aerogel beads proved non-toxic to *D. magna* and biodegradable in soil. This study expands our understanding of biopolymers as alternative adsorbents in wastewater treatment and elucidates important factors in the removal of coexisting pollutants from wastewater.

## 4. Materials and Methods

### 4.1. Materials

SA (C_6_H_7_NaO_6_)n), PVA ((C_2_H_4_O)n, degree of hydrolysis: 86.0–89.0%), calcium chloride dihydrate (CaCl_2_·2H_2_O), copper nitrate trihydrate (Cu(NO_3_)_2_·3H_2_O), hydrochloric acid (HCl, 36%), sodium hydroxide (NaOH), and nitric acid (HNO_3_, 60–62%) were supplied by Daejung Chemicals & Metals (Gyeonggi-do, Korea). CG (C_24_H_36_O_25_S_2_)n was purchased from Tokyo Chemical Industry (Tokyo, Japan). MB dihydrate (C_18_H_18_ClN_3_S·2H_2_O, MW = 355.89) and AB (C_20_H_13_N_2_NaO_5_S, MW = 416.38) were obtained from Showa Chemical (Tokyo, Japan) and Sigma-Aldrich (St. Louis, MO, USA), respectively. Lead nitrate (Pb(NO_3_)_2_) was obtained from Mallinckrodt Chemical Works (St. Louis, MO, USA).

### 4.2. Synthesis of SA-Based Aerogel Beads

**Figure 9:**
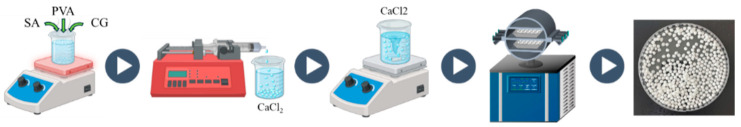
Synthesis procedure of the SA-based aerogel beads.

Four types of SA-based aerogel beads (SA, SA/PVA, SA/CG, and SA/PVA/CG) were synthesized to compare their characteristics and adsorption capacities for Cu(II) and Pb(II). The synthesis procedure was illustrated in Figure 9. Briefly, to synthesize SA aerogel beads, 100 mL of SA (1.5 wt%) solution was prepared using distilled water by dissolving it at 85 °C. For the other formulations, 100 mL solutions of SA/PVA (1:1, 3 wt%), SA/CG (1:1, 2 wt%), and SA/PVA/CG (1:1:1, 3 wt%) were similarly prepared using distilled water. After cooling each solution to room temperature under stirring, the solutions were added drop-wise to a 0.2 M CaCl_2_ solution using a syringe pump (NE-300, New Era Pump Systems Inc., Farmingdale, NY, USA) at a flow rate of 0.3 mL/min. During this step, SA and CG underwent cross-linking. The optimum ratio of SA/PVA/CG (1:1:1) was determined based on its uniform shape, strong cross-linking, and excellent adsorption capacity. The resulting spherical beads were maintained in the CaCl_2_ solution overnight, with stirring at 200 rpm to ensure sufficient cross-linking. The beads were subsequently rinsed with distilled water to remove excess ions, frozen at −20 °C for 24 h, and freeze-dried under vacuum for 48 h. Prior to experiments, the beads were stored in a desiccator.

### 4.3. Comparison of the Characteristics and Adsorption Capacity of the SA-Based Beads

The morphology of the SA, SA/PVA, SA/CG, and SA/PVA/CG beads was observed using field emission scanning electron microscopy (FE-SEM, S-4800, Hitachi, Tokyo, Japan). The porosity, average pore diameter, and pore size range of the beads were assessed using a porosimeter (Autopore V 9620, Micromeritics, Norcross, GA, USA), while the surface functional groups were examined using a Fourier transform infrared spectrophotometer (FTIR, iS-50, Thermo Fisher Scientific, Waltham, WA, USA). The water absorption efficiency of the beads was also evaluated by first soaking the dried beads in distilled water for 24 h and then removing the surface water using tissue paper. The weight of the dried and swollen beads (M_0_ and M_1_, respectively) was used to calculate the water absorption efficiency (%) via Equation (1) [75]:

Water absorption efficiency (%) = (M_1_ − M_0_)/M_0_ × 100 (1)

To compare the adsorption capacity of the SA, SA/PVA, SA/CG, and SA/PVA/CG beads, two heavy metals (Cu(II) and Pb(II)) and a dye (MB) were used in batch experiments. Each type of bead (5 mg) was added to conical tubes containing 20 mL of a Cu(II), Pb(II), or MB solution (initial conc. = 50 mg/L). The mixtures were shaken at 25 °C and 150 rpm in a shaking incubator for 24 h. After the reaction, the samples were filtered using a syringe filter (PVDF, pore size = 0.45 μm) and a 1 mm stainless sieve to separate the beads from the pollutants (Cu(II), Pb(II), or MB). The initial and final concentrations of Cu(II) and Pb(II) were determined using an inductively coupled plasma optical emission spectrometer (ICP-OES, Avio 200, Thermo Fisher Scientific, USA). The adsorption capacity (q_t_) of Cu(II) and Pb(II) was calculated using Equation (2):

q_t_ = ((C_i_ − C_t_) × V/m (2)

where C_i_ (mg/L) and C_t_ (mg/L) are the initial concentrations of Cu(II), Pb(II), or MB and their concentrations at time t, respectively, V (L) is the volume of the solution, and m (g) is the mass of the beads.

The chemical stability of the SA, SA/PVA, SA/CG, and SA/PVA/CG beads was also evaluated under both acidic and basic conditions. The beads were immersed in distilled water adjusted to a pH of 2 or 9 using 0.1 M HCl and 0.1 M NaOH, respectively, for 10 days. The beads were then collected, frozen at −20 °C, and freeze-dried again to examine their morphology using FE-SEM.

### 4.4. Adsorption Mechanisms for the SA/PVA/CG Beads in Single-Pollutant Systems

The effects of the reaction time on the adsorption of the individual pollutants (Cu(II), Pb(II), MB, or AB) onto the SA/PVA/CG beads were evaluated. The SA/PVA/CG beads (5 mg) were mixed with a 20 mL solution of each pollutant (initial conc. = 50 mg/L) and left to react at 25 °C and 150 rpm. Samples were subsequently collected after 0.5, 1, 2, 4, 8, 12, 24, and 48 h. To measure the MB and AB concentrations, the collected samples were filtered through a 1 mm stainless sieve, and the absorbance of the filtrate was measured using a UV–vis spectrophotometer (UV–vis, GENESYS 150, Thermo Fisher Scientific, Waltham, MA, USA) at 665 and 600 nm for MB and AB, respectively. The kinetic data were fitted using pseudo-first-order and pseudo-second-order models, as described in the Supplementary Information (SI).

Adsorption isotherm experiments were conducted with the SA/PVA/CG beads at various initial concentrations of the heavy metal and dye pollutants: 10–125 mg/L for Cu(II) or Pb(II) and 10–500 mg/L for MB. The SA/PVA/CG beads (5 mg) were mixed with 20 mL of each pollutant and left to react at 25 °C and 150 rpm for 48 h. The isotherm data were fitted to the Langmuir, Freundlich, and Temkin models (see the Supplementary Materials for more details).

The effects of the temperature and pH on the adsorption of the individual pollutants (Cu(II), Pb(II), or MB) using SA/PVA/CG beads were also investigated. The SA/PVA/CG beads (5 mg) were added to a 20 mL solution of each pollutant (initial conc. = 50 mg/L) and left for 24 h under temperatures of 288, 298, or 308 K. The Gibb’s free energy (ΔG^0^, kJ/mol), enthalpy (ΔH^0^, kJ/mol), and entropy (ΔS^0^, J/mol∙K) were calculated using the equations listed in the Supplementary Materials). The pH of the initial solution was also adjusted to a range of 2–5 using HCl (0.1 M) and NaOH (0.1 M) solutions. This pH range was selected because the hydroxide precipitation of Cu(II) and Pb(II) occurs above pH 6. The heavy metal species were predicted according to changes in the pH using Visual MINTEQ 4.0. The zeta potential of the SA/PVA/CG beads was measured using a zeta potential analyzer (ZSP, Malvern Instruments, Malvern, UK). XPS analysis was also employed using an X-ray photoelectron spectrometer (K-Alpha+, Thermo Fisher Scientific, USA) before and after the adsorption of the individual pollutants (Cu(II), Pb(II), MB, and AB) onto the SA/PVA/CG beads to determine the adsorption mechanisms. The amount of Ca(II) released after the adsorption of Cu(II) and Pb(II) was measured using ion chromatography (Dionex ICS-5000, Thermo Fisher Scientific, USA).

### 4.5. Adsorption Performance of the SA/PVA/CG Beads Under Binary-Pollutant Conditions

The effect of the reaction time was tested for five binary solutions (Cu–Pb, Cu–MB, Cu–AB, Pb–MB, and Pb–AB) that were prepared by mixing each pollutant at a concentration of 50 mg/L. The SA/PVA/CG beads (5 mg) were added to 20 mL of each binary solution and left to react at 150 rpm and 25 °C for 0.5, 1, 2, 4, 8, 12, 24, and 48 h. To analyze both the heavy metals and the dyes in the same sample, the collected samples were filtered using a PVDF syringe filter (pore size = 0.45 μm), and MB or AB were measured using a UV-vis spectrophotometer. The dye was then completely removed using nitric acid digestion before measuring the Cu(II) and Pb(II) levels using ICP-OES. The results were fitted to pseudo-first-order and pseudo-second-order models to compare them with the data from the single-pollutant systems. The precipitates that formed during the experiments were also analyzed using an SEM–energy dispersive X-ray spectrometer (SEM-EDS, S-4800, Hitachi, Tokyo, Japan).

### 4.6. Acute Toxicity and Biodegradation of the SA/PVA/CG Beads

Acute toxicity testing of the SA/PVA/CG beads was conducted using D. magna (Daphtoxkit F™ magna, MicroBioTests, Gent, Belgium). The experiment process followed OECD guideline 202 [76]. Various doses of the SA/PVA/CG beads (0.03125, 0.0625, 0.125, 0.25, and 0.5 g/L) in 10 mL of standard freshwater were loaded into a multi-well plate with five neonates (age < 24 h) (four replicates for each treatment). The tested neonates were maintained at 20 °C in the dark, and the number of immobilized individuals was recorded after 24 and 48 h of exposure to different doses of the SA/PVA/CG beads. Digital images of D. magna were acquired using an optical microscope (Leica DM500, Leica Microsystems, Wetzlar, Germany) at a magnification of ×100.

To investigate the biodegradability of the SA/PVA/CG beads, the beads were enclosed in nylon fabric bags and buried at a depth of 5 cm in soil. The temperature and humidity were maintained at 25 °C and 70%, respectively, in a SMART Plant Growth Chamber (GC-450, Daihan Scientific Co., Ltd., Wonju, Korea) to ensure consistent conditions. To maintain the moisture levels of the soil, 40 mL of distilled water was added every 7 days for 2 weeks. The weight of the beads and their FT-IR spectra were recorded after 0, 7, and 14 days to monitor the degradation progress of the beads.

## Data Availability

The data presented in this study are available on request from the corresponding author.

## Supplementary Materials

The following supporting information can be downloaded at https://www.mdpi.com/article/10.3390/gels11030211/s1, Figure S1: FE-SEM images of settled beads after chemical stability experiment: (a–c) SA beads in pH 2 and (d–f) pH 9 solution, (g–i) SA/PVA, and (j–l) SA/CG in pH 9 solution; Figure S2: Zeta potential of SA/PVA/CG bead; Figure S3: SEM-EDS spectra of the precipitates formed in the binary system after 24 h reaction: (a) Pb-AB solution; (b) Pb-AB solution with SA/PVA/CG beads; and (c) Cu-AB with SA/PVA/CG beads; Figure S4: FE-SEM images of the SA/PVA/CG aerogel bead after adsorption experiment. Figure S5: XPS spectra of (a) N 1s region after MB adsorption; (b) S 2p of the SA/PVA/CG beads; and (c) S 2p region after MB adsorption; Figure S6: High-resolution of XPS spectra O 1s region before and after adsorption. (a) raw SA/PVA/CG beads; and after single (b) Cu(II), (c) Pb(II), (d) MB adsorption, and (e) Cu-Pb system; Figure S7: Optical microscope images of D. magna after 48 h: (a) control and (b) 5 g/L dose of SA/PVA/CG beads; Figure S8: FTIR spectra of SA/PVA/CG beads before and after biodegradation for 14 days; Table S1: Adsorption kinetic model parameters of SA/PVA/CG beads with single pollutant (Cu(II), Pb(II), and MB); Table S2: Adsorption isotherm model parameters of the SA/PVA/CG beads for the adsorption of Cu(II), Pb(II), and MB; Table S3: Comparison of the adsorption capacities of Cu(II), Pb(II), and MB on SA, PVA, and CG based adsorbents; Table S4: Thermodynamic parameters of the SA/PVA/CG beads (dose = 0.25 g/L) for the adsorption of Cu(II), Pb(II), or MB (initial conc. = 50 mg/L); Table S5: Single and binary pollutant adsorption kinetic model parameters for the adsorption of Cu(II), Pb(II), MB, and AB using the SA/PVA/CG beads; Table S6: SEM-EDS analysis of precipitates on binary system.
