# Supplementary material for: Simultaneous Removal of Heavy Metals and Dyes on Sodium Alginate/Polyvinyl Alcohol/κ-Carrageenan Aerogel Beads
Source: Gels. 2025 Mar 16;11(3):211. doi: 10.3390/gels11030211 (PMC11942351; doi:10.3390/gels11030211)
Supplement: Supplementary file 1 [file gels-11-00211-s001.zip › gels-3533145-supplementary.pdf]

Simultaneous Removal of Heavy Metals and Dyes on Sodium Alginate/Polyvinyl Alcohol/κ-Carrageenan Aerogel Beads

Taesoon Jang, Soyeong Yoon, Jin-Hyuk Choi, Narae Kim and Jeong-Ann Park *

Department of Environmental Engineering, Kangwon National University, Chuncheon 24341, Republic of Korea; zxotnsflz@kangwon.ac.kr (T.J.); sy3436@kangwon.ac.kr (S.Y.); cjh0722@kangwon.ac.kr (J.-H.C.); narae345@kangwon.ac.kr (N.K.)

* Correspondence: pjaan@kangwon.ac.kr; Tel.: +82-33-250-6359

The kinetic data were fitted using pseudo-first-order (Eq. 7), pseudo-second-order (Eq. 8) models, respectively.

$$q_t = q_e(1 - e^{-k_1 t}) \tag{7}$$

$$q_t = \frac{k_2 q_e^2 t}{1 + q_e k_2 t} \tag{8}$$

Where q_t and q_e (mg/g) are the adsorption capacity at time t (h) and equilibrium. k_1 (/h), k_2 (g/mg/h), and k_{id} (mg/g/h$^{0.5}$) represent the rate constant of each model. C (mg/g) is the intercept constant at the initial stage of the adsorption process, which represents the thickness of the boundary layer.

The isotherm data were fitted to the Langmuir (Eq. 4), Freundlich (Eq. 5), and Temkin (Eq. 6) models.

$$q_e = q_m \frac{K_L C_e}{1 + K_L C_e} \tag{4}$$

$$q_e = K_F C_e^{1/n} \tag{5}$$

$$q_e = \frac{RT}{b_T}\ln(A_T C_e) \quad (6)$$

Where q_e(mg/g) and q_m (mg/g) is the equilibrium and maximum adsorption capacity respectively. K_L (L/mg) and K_F (L/g) are Langmuir and Freundlich constant, respectively. *1/n* represents the Freundlich constant related to the adsorption intensity. R (J/mol·K) is ideal gas constant. b_T (J/mol) is the adsorption heat constant and A_T (L/g) is the equilibrium binding constant.

To verify the effect of temperature, different temperatures varied at 288, 298, and 308 K. The thermodynamic parameters such as Gibb's free energy (ΔG^0, kJ/mol), enthalpy (ΔH^0, kJ/mol), and entropy (ΔS^0, J/mol·K) were calculated by following Eqs. (10–12).

$$K_e = \frac{q_e}{C_e} \quad (10)$$

$$\Delta G^\circ = -RTlnK_e \quad (11)$$

$$\ln(K_e) = \frac{\Delta S^\circ}{R} - \frac{\Delta H^\circ}{RT} \quad (12)$$

Where K_e is the equilibrium constant, T (K) is the temperature, and R (J/mol·K) is the ideal gas constant.

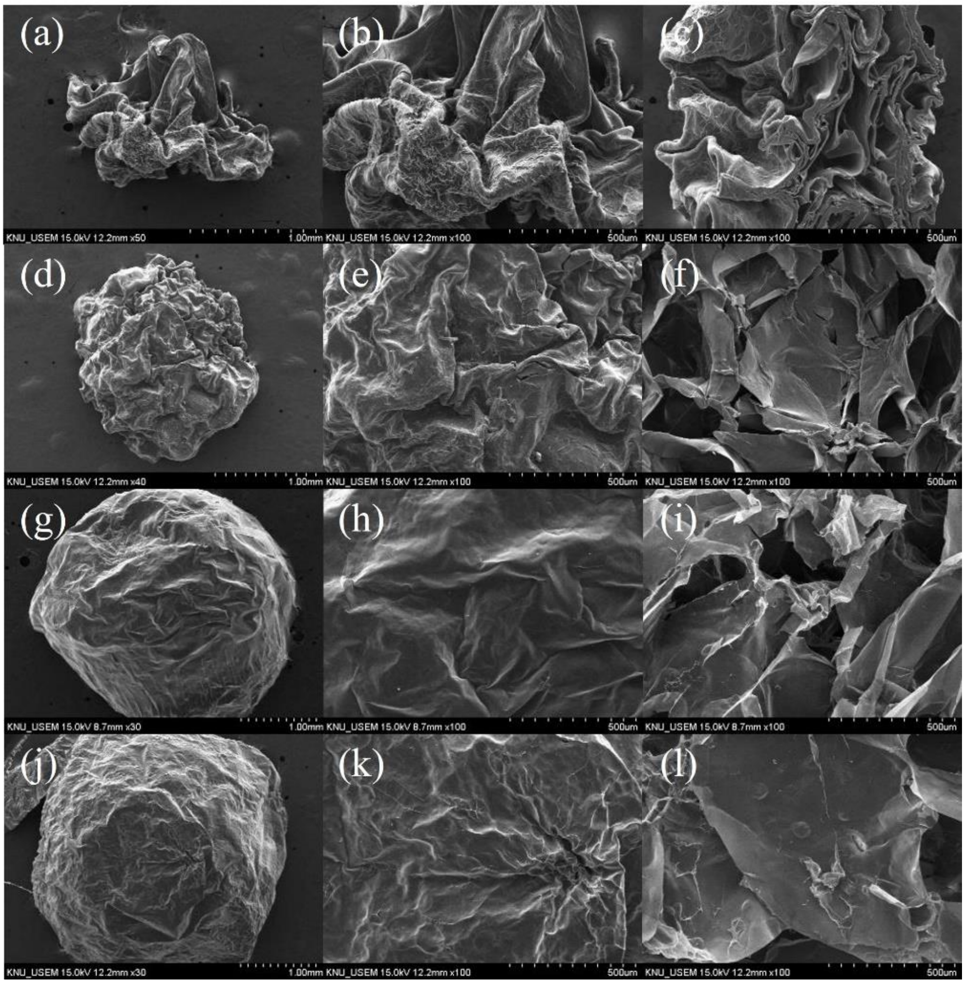

Fig. S1. FE-SEM images of settled beads after chemical stability experiment: (a-c) SA beads in pH 2 and (d-f) pH 9 solution, (g-i) SA/PVA, and (j-l) SA/CG in pH 9 solution.

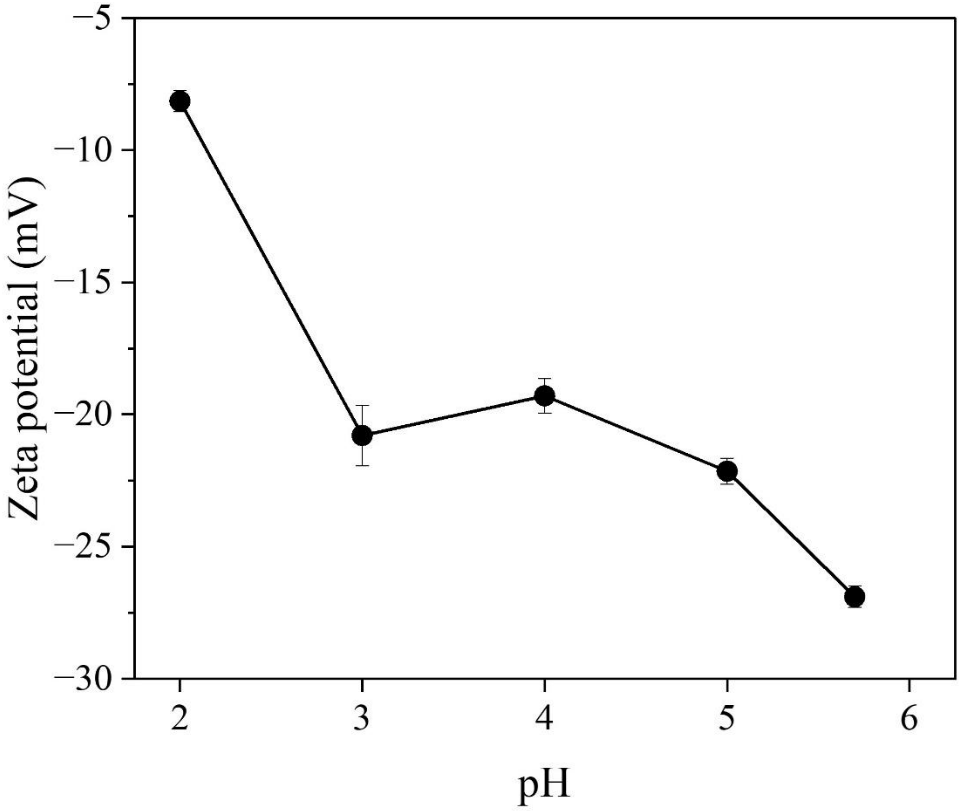

Fig. S2. Zeta potential of SA/PVA/CG bead.

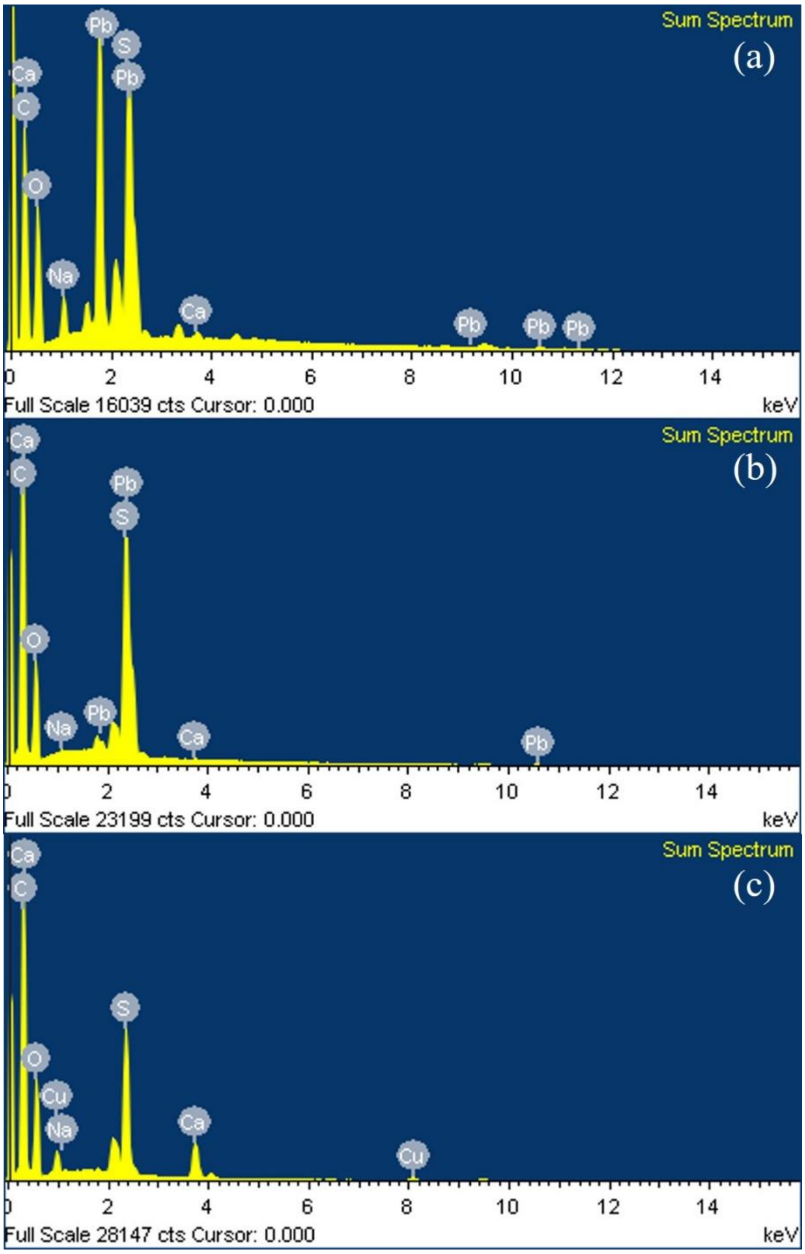

Fig. S3. SEM-EDS spectra of the precipitates formed in the binary system after 24 h reaction: (a) Pb-AB solution; (b) Pb-AB solution with SA/PVA/CG beads; and (c) Cu-AB with SA/PVA/CG beads

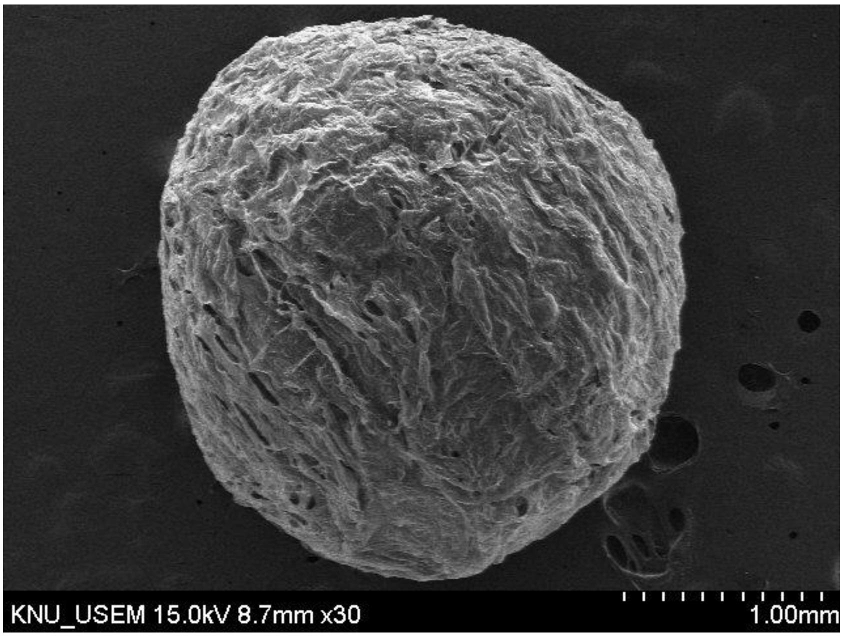

Fig. S4. FE-SEM images of the SA/PVA/CG aerogel bead after adsorption experiment.

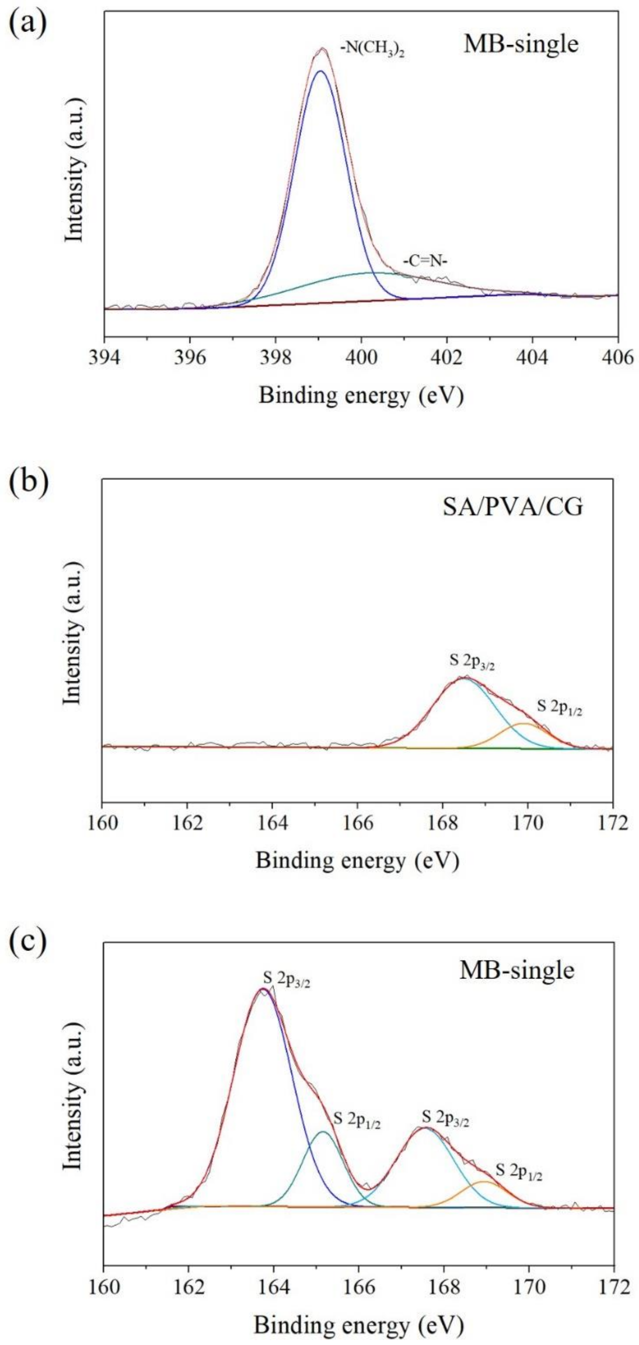

Fig. S5. XPS spectra of (a) N 1s region after MB adsorption; (b) S 2p of the SA/PVA/CG beads; and (c) S 2p region after MB adsorption.

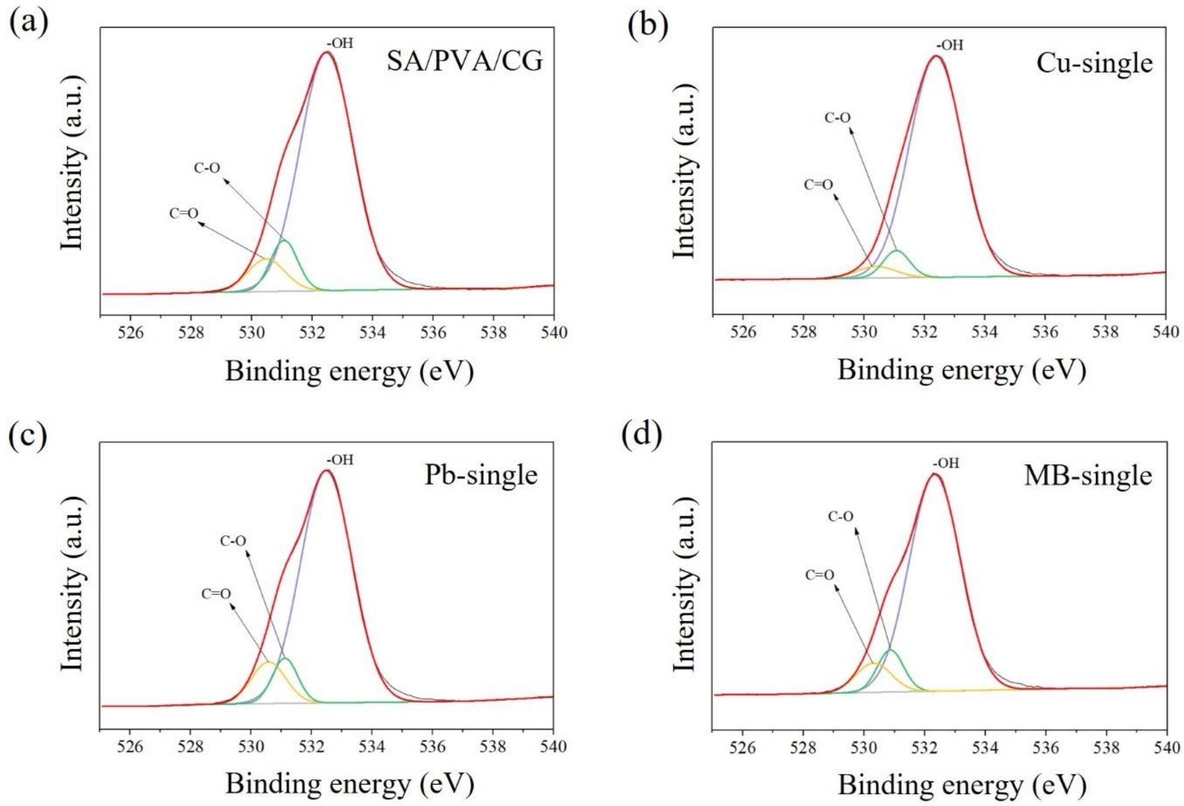

Fig. S6. High-resolution of XPS spectra O 1s region before and after adsorption. (a) raw SA/PVA/CG beads; and after single (b) Cu(II), (c) Pb(II), (d) MB adsorption, and (e) Cu-Pb system.

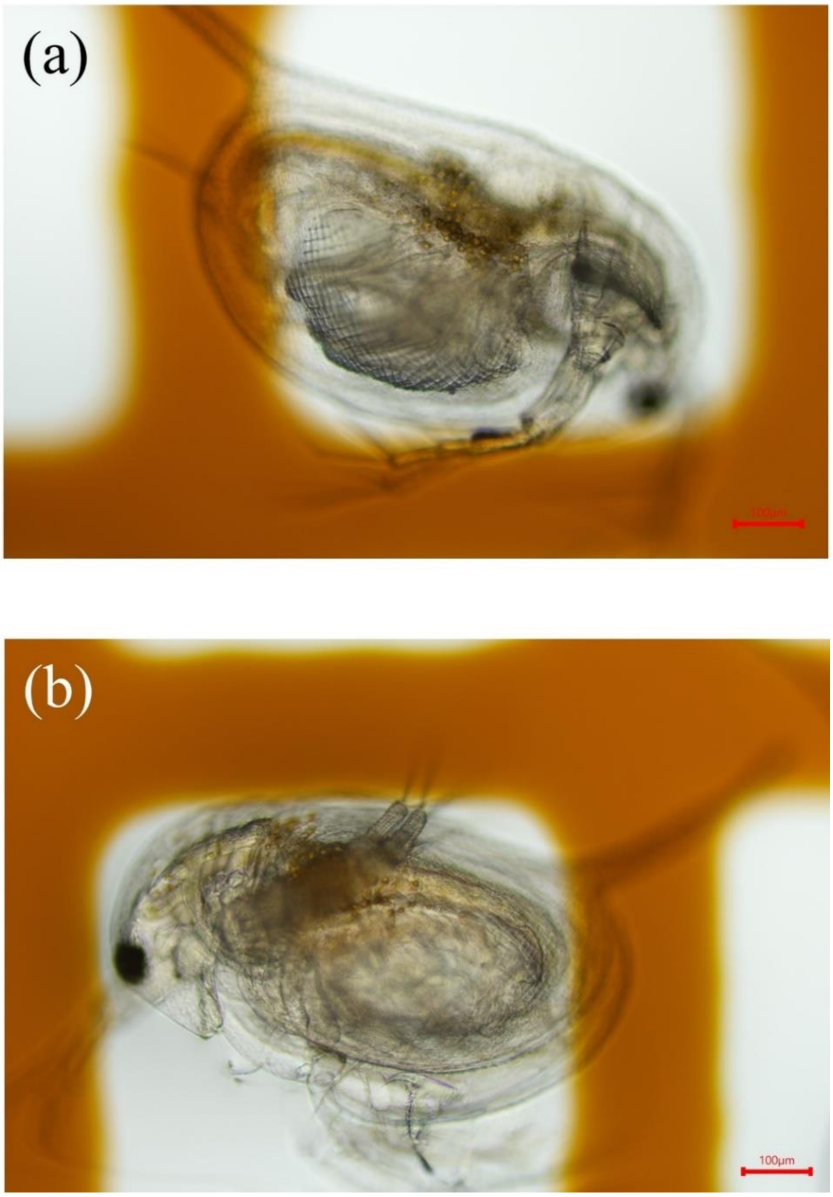

Fig. S7. Optical microscope images of *D. magna* after 48 h: (a) control and (b) 5 g/L dose of SA/PVA/CG beads.

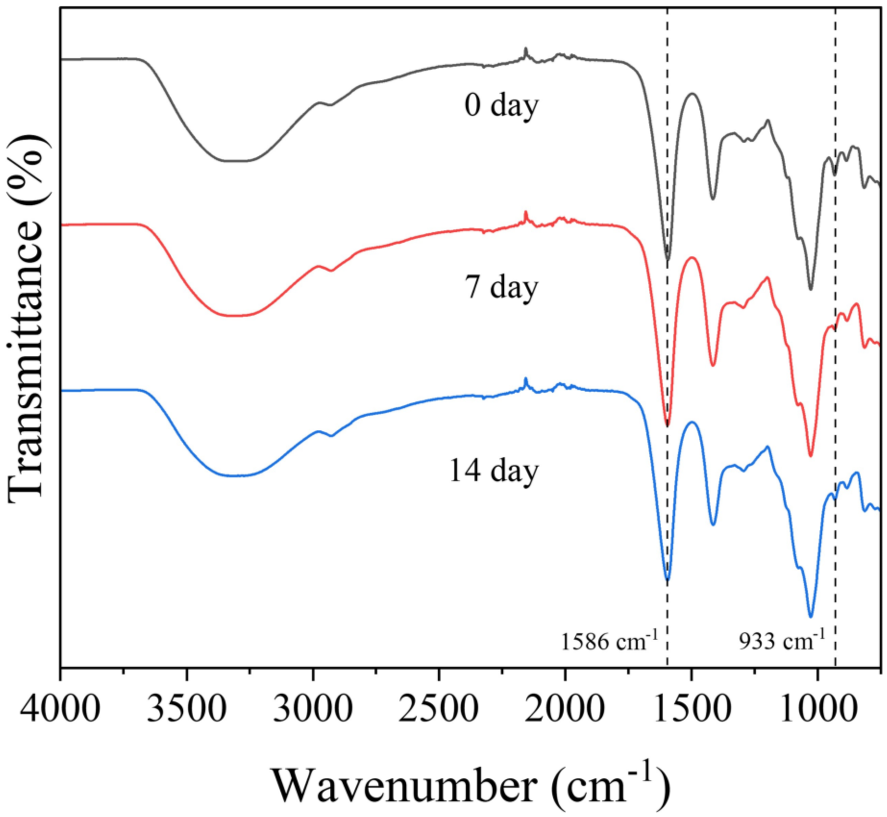

Fig. S8. FTIR spectra of SA/PVA/CG beads before and after biodegradation for 14 days.

Table S1

Adsorption kinetic model parameters of SA/PVA/CG beads with single pollutant (Cu(II), Pb(II), and MB).

Bead	Pollutant	Psuedo-first-order			Psuedo-second-order		
		k_1 (/h)	q_e (mg/g)	R^2	k_2 (g/mg/h)	q_e (mg/g)	R^2
SA/PVA/CG	Cu(II)	1.34	74.95	0.948	0.03	73.29	0.989
	Pb(II)	1.60	177.89	0.969	0.01	187.28	0.998
	MB	0.98	190.46	0.912	0.01	204.53	0.873

Table S2

Adsorption isotherm model parameters of the SA/PVA/CG beads for the adsorption of Cu(II), Pb(II), and MB.

Pollutant	Langmuir			Freundlich			Temkin		
	q_m (mg/g)	K_L (L/mg)	R^2	K_F (L/g)	1/n (-)	R^2	b_T (kJ/mol)	A_T (L/g)	R^2
Cu(II)	85.17	0.43	0.938	38.53	0.18	0.946	0.21	16.84	0.959
Pb(II)	265.98	1.27	0.968	123.27	0.22	0.936	0.06	27.21	0.972
MB	1324.30	0.035	0.998	139.26	0.40	0.937	0.01	0.93	0.876

Table S3

Comparison of the adsorption capacities of Cu(II), Pb(II), and MB on SA, PVA, and CG based adsorbents.

Adsorbent	Pollutant	pH	Temperature (°C)	Dose of adsorbent (g/L)	q_m (mg/g)	Ref.
PVA/SA	MB	-	25	1	100.36 (C_0 = 50-400)	Lin et al. (2022)
PVA/SA/GO	MB	-	25	1	189.78 (C_0 = 50-400)	Lin et al. (2022)
Zeo/PVA/SA NC	Cu	-	25	20	48.544 (C_0 = 10-100)	Isahi (2020)
Zeo/PVA/SA NC	Pb	-	25	20	47.619 (C_0 = 10-100)	Isahi (2020)
SA bead	Cu	5	room temperature	1	29.19 (C_0 = 20-500)	[53]
PVA/SA bead	Cu	5	room temperature	1	34.31 (C_0 = 20-500)	[53]
PVA/SA@PAM bead	Cu	5	room temperature	1	70.45 (C_0 = 20-500)	[53]
SAGO aerogel	Cu	5	30	-	98.0 (C_0 = 25-600)	[50]
SAGO aerogel	Pb	5.5	-	-	267.4 (C_0 = 25-600)	[50]
SA-PAVB/GO	Cu	5	25	1	105.93 (C_0 = 25-250)	[51]
SA-PAVB/GO	Pb	5.5	25	1	191.94 (C_0 = 50-500)	[51]
SA hydrogel	MB	5.5	room temperature	0.5	94.3 (C_0 = 10-400)	[55]
SA hydrogel	MB	5.5	room temperature	0.5	94.3 (C_0 = 10-400)	[55]
SA/PEI hydrogel	MB	5.5	room temperature	0.5	400.0 (C_0 = 10-400)	[55]

Table S3

(Continued).

Adsorbent	Pollutant	pH	Temperature (°C)	Dose of adsorbent (g/L)	q_m (mg/g)	Ref.
PVA-SA–CTS-montmorillonite hydrogel beads	MB	8	30	2	137.15 (C_0 = 10-70)	Wang et al. (2018)
GO/SA hydrogel membrane	Pb	5.0	30	-	284.9 (C_0 = 5-80)	[54]
GO/SA hydrogel membrane	Pb	5.0	45	-	327.9 (C_0 = 5-80)	[54]
modified magnetic CG (MMCG)	Cu	6	-	1	114.94 (C_0 = 20-200)	[52]
modified magnetic CG (MMCG)	Pb	6	-	1	120.48 (C_0 = 20-200)	[52]
k-Car/GO gel bead	MB	5.25	25	50 beads to 20 mL	628.93 (C_0 = 100-800)	[56]
PVA/CG membrane	MB	-	25	3.33	147.8 (C_0 = 10-500)	Radoor et al. (2024)
CTS/PVA	Cu	6	-	0.2	47.85 (C_0 = 0-14)	Ngah (2008)
CTS/PVA/PEG bead	Cu	5	45	1	44.05 (C_0 = 10-200)	Trikkaliotis et al. (2020)
Macroreticular PVA bead	Pb	6	15	1	213.98 (C_0 = 10-500)	Zhang et al. (2010)
This study	Cu	5	25	0.25	85.17 (C_0 = 10-125)	This study
This study	Pb	5	25	0.25	265.98 (C_0 = 10-125)	This study
This study	MB	5	25	0.25	1324.30 (C_0 = 10-500)	This study

Table S4

Thermodynamic parameters of the SA/PVA/CG beads (dose = 0.25 g/L) for the adsorption of Cu(II), Pb(II), or MB (initial conc. = 50 mg/L).

Temperature (K)	Cu(II)			Pb(II)			MB		
	ΔG^0 (KJ/mol)	ΔH^0 (KJ/mol)	ΔS^0 (J/mol·K)	ΔG^0 (KJ/mol)	ΔH^0 (KJ/mol)	ΔS^0 (J/mol·K)	ΔG^0 (KJ/mol)	ΔH^0 (KJ/mol)	ΔS^0 (J/mol·K)
288	-1.96	4.48	22.36	-10.16	9.79	69.29	-9.64	-27.78	-62.95
298	-2.18			-10.86			-9.02		
308	-2.41			-11.55			-8.38		

Table S5

Single and binary pollutant adsorption kinetic model parameters for the adsorption of Cu(II), Pb(II), MB, and AB using the SA/PVA/CG beads.

Pollutant	System	Pseudo-first-order			Pseudo-second-order		
		k_1 (/h)	q_e (mg/g)	R^2	k_2 (g/mg/h)	q_e (mg/g)	R^2
Cu(II)	Cu	1.34	74.95	0.948	0.03	79.29	0.989
	Cu-Pb	2.16	35.27	0.949	0.11	36.87	0.980
	Cu-MB	0.78	72.38	0.900	0.01	78.51	0.969
	Cu-AB	0.48	91.96	0.964	0.01	100.99	0.990
Pb(II)	Pb	1.60	177.89	0.969	0.01	187.28	0.998
	Cu-Pb	1.44	138.27	0.918	0.02	147.33	0.975
	Pb-MB	1.04	156.74	0.899	0.01	168.90	0.967
	Pb-AB	3.57	184.94	0.988	0.05	189.57	0.998
MB	MB	0.98	190.46	0.912	0.01	204.53	0.873
	Cu-MB	0.53	111.61	0.788	0.01	125.78	0.893
	Pb-MB	0.44	125.63	0.799	0.00	140.25	0.901

Table S6

SEM-EDS analysis of precipitates on binary system.

Solution	Addition of bead	Weight (%)						
		C	O	Na	S	Ca	Cu	Pb
Cu-AB	Yes	59.61	24.40	-	9.03	3.78	3.27	-
Pb-AB	Yes	53.21	19.94	0.12	7.11	0.08	-	19.52
Pb-AB	No	41.03	24.30	1.91	8.80	0.66	-	23.30
